# Association of genitourinary infections and cervical length with preterm childbirth

**DOI:** 10.1590/1414-431X202010235

**Published:** 2020-12-21

**Authors:** F.M.M. Bernardo, E.C.A. Veiga, S.M. Quintana, F.J.A. Camayo, R.F.L. Batista, M.T.S.S.B. Alves, H. Bettiol, M.A. Barbieri, V.C. Cardoso, R.C. Cavalli

**Affiliations:** 1 Departamento de Ginecologia e Obstetrícia, Faculdade de Medicina de Ribeirão Preto, Universidade de São Paulo, Ribeirão Preto, SP, Brasil; 2 Departamento de Puericultura e Pediatria, Faculdade de Medicina de Ribeirão Preto, Universidade de São Paulo, Ribeirão Preto, SP, Brasil; 3 Departamento de Saúde Pública, Universidade Federal do Maranhão, São Luís, MA, Brasil

**Keywords:** Preterm birth, Prematurity, Genital infection, Urinary infection, Uterine cervix

## Abstract

A prospective cohort study was conducted on a convenience sample of 1370 pregnant women with a gestational age of 20 to 25 weeks in the city of Ribeirão Preto. Data on obstetrical history, maternal age, parity, smoking habit, and a history of preterm delivery was collected with the application of a sociodemographic questionnaire. Cervical length was determined by endovaginal ultrasound, and urine and vaginal content samples were obtained to determine urinary tract infection (UTI) and bacterial vaginosis (BV), respectively. The aim of this study was to verify the association of cervical length and genitourinary infections with preterm birth (PTB). Ultrasound showed no association of UTI or BV with short cervical length. PTB rate was 9.63%. Among the women with PTB, 15 showed UTI (RR: 1.55, 95%CI: 0.93–2.58), 19 had BV (RR: 1.22, 95%CI: 0.77–1.94), and one had both UTI and BV (RR: 0.85, 95%CI: 0.13–5.62). Nineteen (14.4%) PTB occurred in women with a cervical length ≤2.5 cm (RR: 2.89, 95%CI: 1.89–4.43). Among the 75 patients with PTB stratified as spontaneous, 10 showed UTI (RR: 2.02, 95%CI: 1.05–3.86) and 14 had a diagnosis of BV (RR: 1.72, 95%CI: 0.97–3.04). A short cervical length between 20 and 25 weeks of pregnancy was associated with PTB, whereas UTI and BV determined at this age were not associated with short cervical length or with PTB, although UTI, even if asymptomatic, was related to spontaneous PTB.

## Introduction

Preterm birth (PTB) is an important public health problem due to its high incidence and perinatal morbidity-mortality (1). Brazilian studies have reported a prevalence of PTB ranging from 11.7 to 12.3% (2). PTB and its complications are responsible for 78% of all neonatal deaths and are associated with high morbidity, with serious short- and long-term consequences involving physical, psychological, and economic costs (3,4).

Studies have demonstrated that infections such as bacterial vaginosis (BV) and urinary tract infections (UTI) may be associated with a higher risk of PTB and low birth weight (5,6), since these processes are related to the inflammatory response present in infections. These conditions are fundamentally involved in the physiopathology of PTB since cytokines (TNF-α, interleukins, and prostaglandins - PGE2, PGD2, and PGF2α, in particular) resulting from the cascade of inflammatory events that directly participate in the triggering of uterine contractions (7,8).

Cervical effacement causing cervical shortening precedes by five to six weeks the clinically determined labor (9,10). Thus, the identification of cervical shortening at early gestational ages represents an important risk factor for PTB (11–13) and its evaluation by transvaginal ultrasound is one of the parameters showing a good correlation with the risk for PTB (14).

On this basis, our objective was to assess the association of genitourinary infections with cervical shortening and their relationship with previous PTB (PPTB).

## Material and Methods

The study was approved by the Research Ethics Committee of the University Hospital, Faculty of Medicine of Ribeirão Preto, University of São Paulo (HC-FMRPUSP) in 2008 (protocol No. 11157/2008), and all women gave written informed consent to participate.

This case-control study was nested in a prospective convenience cohort investigated in 2010 and 2011. The cohort has been described in the thematic project entitled "Ecological factors of preterm birth and consequences of perinatal factors on children’s health: birth cohorts in two Brazilian cities - BRISA" (https://nesca.fmrp.usp.br/brisa-botoes) conducted in Ribeirão Preto (SP) and São Luís (MA). The Ribeirão Preto data were used in the present study.

In this study, we used a convenience sample from the BRISA thematic project whose size was based on the reported prevalence of the explanatory variables studied in the initial project, which varied from 10 to 50%. Thus, considering a 12% prematurity rate, we started from the initial sample of 1500 women and their respective newborns. Considering losses and lack of information, the sample effectively consisted on 1370 mother-child pairs and a total of 132 PTB. Of all PTB, we were able to obtain information about the characteristics of childbirth, spontaneous or not, for 102 participants.

We included pregnant women residing in Ribeirão Preto, seen in public and private services, carrying a single fetus, and evaluated during prenatal care at a gestational age of 20 to 25 weeks. The criteria for inclusion in the prenatal BRISA cohort were: having done an obstetrical ultrasound exam before the 20th week of gestation, having a gestational age of 20 to 25 weeks at the time of data collection, and having a single fetus.

Women who were lost to follow-up between the prenatal and birth evaluations and women with incomplete information were excluded from the study. Gestational age was estimated from the date of last menstrual period (DLM) and based on the gestational ultrasound performed before 20 weeks of pregnancy, thus minimizing determination bias in the estimate of outcome (15).

After responding to a standardized questionnaire in a face-to-face interview, the women were submitted to gynecological examination and collection of vaginal material for fresh examination and for the preparation of Gram-stained slides. The criterion used for the diagnosis of BV was the presence of clue cells and/or a Nugent score with counts of existing *Lactobacillus*, *Gardnerella*/*Bacteroides*, and *Mobiluncus* morphotypes. Morphological types where scored as 1+ to 4+ according to the number of microorganisms present. A score of seven or more was considered to indicate positivity for BV (16–18). Urine was collected for culture, considered the gold standard method validated for the investigation of asymptomatic bacteriuria and the detection of UTI (19).

Ultrasound exams were performed at the Clinical Research Unit of FMRP-USP by trained observers calibrated for the method, using an HDI instrument, model 11 (USA), and a technique systematized and validated by the Fetal Medicine Foundation (20). Controls were obtained from the cases that did not progress to PTB, at a 2:1 proportion (21). In view of the well-established association of short cervix with prematurity, all patients with a cervical length of 2.5 cm or less were referred to a center specialized in high-risk pregnancies.

After birth, newborn data regarding hospital, weight, and gestational age were obtained and a standardized puerperium questionnaire was applied in order to obtain childbirth information about the 95 participants, with the analysis of replies such as presence of painful contractions preceding the outcome in order to determine whether labor was spontaneous. Participants whose questionnaires did not contain sufficient information were contacted by telephone and 7 of them were located. The difference between groups was analyzed by the Fisher exact test, and the relative risk of the various variables was calculated by the adjustment of log-binomial models and confidence intervals (95%CI) using the SAS 9.2 software (USA).

The variables were defined according to their relationship with risk of PTB: cervical length (≤2.5 or >2.5 cm); presence or absence of urinary infection and/or BV; age (<19 years, 19 to 35 years, and >35 years; parity (1, 2, 3, 4, or more children); smoking (yes or no); previous preterm birth (yes or no) (22–25).

## Results

The general characteristics of the study population are shown in Table 1. The study was conducted on 1370 pregnant women, 132 (9.63%) of whom had PTB. Among these 132 patients with PTB, information about childbirth was available for 102, with a total of 74 spontaneous PTB and 28 nonspontaneous PTB. Of the 74 women with spontaneous PTB, 10 had UTI and 14 had BV (data not shown in a table).

**Table 1:** Details about the population studied: maternal age, PPTB (previous preterm birth), race, schooling, and cervical length.

General characteristics	
Age	
<19	11.78%
19−35	80.92%
>35	7.2%
PPTB	
None	85%
1	11.9%
2 or more	2.1%
Race	
White	31.7%
Black	68.3%
Schooling	
Illiterate, elementary school incomplete	2.3%
Elementary school complete, middle school incomplete	14.1%
Middle school complete, high school incomplete	75.1%
High school complete, higher education incomplete	3.6%
Higher education complete	4.5%
Cervical length	
Median	3.5 cm

The Fisher exact test was applied to determine the association between the presence of genitourinary infections and a short cervix in an ultrasound exam. A cervical length of 2.5 cm or less was observed in 7 (5.79%) of 121 patients with a positive UTI exam and in 68 (5.46%) of the 1245 patients with no UTI (P=0.83). A cervical length of 2.5 cm or less was observed in 13 (7.07%) of 184 patients with a positive BV exam and in 61 (5.21%) of those with no BV, again without statistical significance (P=0.30). Among the 13 patients with a positive exam for the two infections, no cervical length ≤2.5 was observed.

Regarding the infections, patients with UTI had a crude RR of 2.02 with 95%CI: 1.05–3.863 and an adjusted RR of 2.205 with 95%CI: 1.109–4.385, whereas patients with BV had a crude RR of 1.72 with 95%CI: 0.97–3.04 and an adjusted RR of 1.2 with 95%CI: 0.64–2.24. No patient had association of the two infections. Regarding the cervix (see details in Table 2), in the group of women with a cervical length ≤2.5 cm, the crude RR was 4.11 with 95%CI: 2.42-7.00, and adjusted RR was 2.66 with 95%CI: 1.46–4.87.

**Table 2:** Correlations between spontaneous preterm birth (PTB) and cervix ≤2.5 cm, infection, age, parity, smoking, and previous PTB.

	Spontaneous PTB		P value	Crude RR	95%CI
	No (n=1214)	Yes (n=74)			
Cervix ≤2.5					
No	1156 (95.38)	59 (80.82)	Ref	Ref	Ref
Yes	56 (4.62)	14 (19.18)	≤0.0001	4.119	2.423-7.001
Infection					
None	971 (79.98)	50 (67.57)	Ref	Ref	Ref
Vaginosis	152 (12.52)	14 (18.92)	0.061	1.722	0.974-3.044
UTI	91 (7.5)	10 (13.51)	0.033	2.022	1.058-3.863
Age					
<19	144 (11.86)	10 (13.51)	0.640	1.168	0.610-2.236
19 to 35	985 (81.14)	58 (78.38)	Ref	Ref	Ref
>35	85 (7)	6 (8.11)	0.681	1.186	0.526−2.673
Parity					
1	530 (43.66)	29 (39.19)	0.678	0.902	0.556-1.466
2 and 3	541 (44.56)	33 (44.59)	Ref	Ref	Ref
4 or more	143 (11.78)	12 (16.22)	0.83	1.347	0.713-2.455
Smoking					
Yes	277 (22.82)	27 (36.49)	0.008	1.859	1.179-2.932
No	937 (77.18)	47 (63.51)	Ref	Ref	Ref
Previous PTB					
None	1105 (91.25)	22 (29.73)	Ref	Ref	Ref
1	85 (7.02)	42 (56.76)	≤0.0001	16.942	10.461-27.437
2 or more	21 (1.73)	10 (13.51)	≤0.0001	16.525	8.568-31.871

Data are reported as number and percentage. The difference between groups was analyzed by the Fisher exact test and the relative risk of the various variables was calculated by the adjustment of log-binomial models and confidence intervals (95%CI). UTI: urinary tract infection.

Taking into account previous preterm births (PPTB), for patients with 1 PPTB, the crude RR was 16.94 (95%CI: 10.46–27.43) and the adjusted RR was 15.83 (95%CI: 9.37–26.74). For patients with 2 or more PPTB, the crude RR was 16.52 (95%CI: 8.56–31.87) and the adjusted RR was 12.83 (95%CI: 5.97 to 27.56). Patients who reported that they were smokers had a crude RR of 1.85 (95%CI: 1.17–2.93) and an adjusted RR of 1.31 (95%CI: 0.79–2.18). The reference for this calculation was the group of nonsmokers. Regarding parity, primiparous patients had a crude RR of 0.90 (95%CI: 0.55–1.46); the adjusted RR could not be calculated since a history of PTB was one of the variables that did not apply to this group. Patients in their second or third pregnancy were the reference used for this calculation. The group of patients with 4 or more pregnancies had a crude RR of 1.34 (95%CI: 0.71–2.45). Regarding age, pregnant women younger than 19 years had a crude RR of 1.16 (95%CI: 0.61–2.23) and the adjusted RR could not be calculated. The group aged 19 to 35 years was used as reference for the calculations. Patients older than 35 years had a crude RR of 1.18 (95%CI: 0.52–2.67) and the adjusted RR could not be calculated. Details are presented in Table 3 and Table 4.

**Table 3:** Crude and adjusted relative risk of spontaneous preterm birth (PTB) (and confidence intervals) associated with infection and cervical length.

	Spontaneous PTB		Crude RR (95%CI)	Adjusted RR (95%CI)
	Yes (n=74)	No (n=1214)		
Infection				
None	50 (67.57)	971 (79.98)	Ref	Ref
UTI	10 (13.51)	91 (7.5)	2.02 (1.05-3.86)	2.20 (1.10-4.38)
BV	14 (18.92)	152 (12.52)	1.72 (0.97-3.04)	1.20 (0.64-2.24)
Cervix <2.5 cm				
No	59 (80.82)	1156 (95.38)	Ref	Ref
Yes	14 (19.18)	56 (4.62)	4.11 (2.42-7.00)	2.66 (1.46-4.87)

Data are reported as number and percentage. CI: confidence interval; RR: relative risk; UTI: urinary tract infection; BV: bacterial vaginosis; Ref: Reference for the calculation. To calculate the crude RR, univariate analysis was performed. To calculate the adjusted RR, multivariate analysis was used with the variables that in the univariate showed an association with the outcome. The adjusted RR was corrected for the following variables: infection, cervical length, previous PTB, smoking habit, parity, and age.

**Table 4:** Crude and adjusted relative risk of spontaneous preterm birth (PTB) (and confidence intervals) associated with previous PTB, smoking habit, parity, and age.

	Spontaneous PTB		Crude RR (95%CI)	Adjusted RR
	Yes	No		
Previous PTB				
None	22 (29.73)	1105 (91.25)	Ref	Ref
1	42 (56.76)	85 (7.02)	16.94 (10.46-27.43)	15.83 (9.37-26.74)
2 or more	10 (13.51)	21 (1.73)	16.52 (8.56-31.87)	12.83 (5.97-27.56)
Smoking habit				
Yes	27 (36.49)	277 (22.82)	1.85 (1.17-2.93)	1.31 (0.79-2.18)
No	47 (63.51)	937 (77.18)	Ref	Ref
Parity				
1	29 (39.19)	530 (43.66)	0.90 (0.55-1.46)	
2 and 3	33 (44.59)	541 (44.56)	Ref	
4 or more	12 (16.22)	143 (11.78)	1.34 (0.71−2.45)	
Age				
<19	10 (13.51)	144 (11.86)	1.16 (0.61−2.23)	
19-35	58 (78.38)	985 (81.14)	Ref	
>35	6 (8.11)	85 (7)	1.18 (0.52−2.67)	

Data are reported as number and percentage. CI: confidence interval; RR: relative risk; Ref: Reference for the calculation. To calculate the crude RR, univariate analysis was performed. To calculate the adjusted RR, multivariate analysis was used with the variables that in the univariate analysis showed an association with the outcome. The adjusted RR was corrected for the following variables: infection, cervical length, previous PTB, smoking habit, parity, and age.

## Discussion

The present study revealed a 9.63% incidence of PTB, a value below that reported in the literature. In Brazil, a 2010 study coordinated by the Postgraduate Program of Epidemiology of the Federal University of Pelotas with the participation of 12 universities, reported that 11.7% of the births occurring in Brazil are preterm (26). The 2014 Brazilian Multicenter Study on Preterm Birth (EMIP), involving 20 reference centers distributed throughout Brazil and data obtained from April 2011 to July 2012, pointed out that 12.3% of the births were preterm (27). The lower rate of PTB in our study could be due to the referral of patients diagnosed with a short cervix to a prematurity center, reducing the rate of prematurity.

Data analysis revealed that cervical shortening was not associated with the occurrence of infections (UTI and BV) between 20 and 25 weeks of pregnancy. The hypothesis of such association was raised because infections, as well cervical shortening, are associated with PTB. We considered the immuno-inflammatory theory of PTB and attempted to determine whether the cytokines and prostaglandins produced in inflammatory and infectious processes, which trigger uterine contractions and depolymerization of the cervix, were linked to cervical shortening, as proposed by Romero et al. (5), Hanna et al. (7), and Chalis et al. (8). We wish to point out that the infections of the women studied here were asymptomatic and might have released lower amounts of inflammatory mediators, thus having a lower influence on cervical size.

A cervical length ≤2.5 cm determined between 20 and 25 weeks of gestation was associated with PTB, supporting data obtained by others. In 1966, Schaffner and Schanzer (9) were the first to report the association of cervical shortening with PTB and many important studies have been conducted since then showing this association. An example is a study by Iams et al. (12) who investigated 2915 gestations of approximately 24 weeks and detected the following associations with PTB: RR of 3.79 (95%CI: 2.32–6.19) for a cervical length of less than 30 mm, RR of 6.19 (95%CI: 3.84–9.97) for a length of less than 26 mm, RR of 9.49 (95%CI: 5.95–15.15) for a length of less than 22 mm, and RR of 13.99 (95%CI: 7.89–24.78) for a length of ≤2.5 cm. In a prospective multicenter observational study conducted from 1998 to 2006, Celik el al. (20) determined the cervical length of 58,807 women between 20 and 25 weeks of pregnancy and demonstrated a higher risk of PTB when cervical length was less than 26 mm (RR of 1.05 for PTB <28 weeks; 1.93, between 28 and 30 weeks; 1.95, between 31 and 33 weeks, and 1.44, between 34 and 36 weeks). Regarding infections, there was only an association of UTI with spontaneous PTB. The presence of asymptomatic bacteriuria screened by urine culture between 20 and 25 weeks of pregnancy was significantly associated with an increased risk of spontaneous PTB (RR 2.2, 95%CI: 1.1–4.4).

Of 199,093 patients studied by Scheiner et al. (28), 4890 (2.5%) showed asymptomatic bacteriuria, 13.3% of whom had PTB, as opposed to 7.6% of patients with a negative exam (OR 1.9, 95%CI: 1.7–2.0), demonstrating the increased risk of pregnant women with asymptomatic bacteriuria.

The determination of BV between 20 and 25 weeks of gestation was not associated with PTB in our study. A meta-analysis of 32 studies with a total of 30,518 patients published by Leitich and Kiss (29) revealed a significant increase in PTB among women with symptomatic or asymptomatic BV diagnosed by clinical criteria or by Gram exams at various gestational ages. Klebanoff et al. (30) compared the risk of PTB in 12,937 women with asymptomatic or negative BV evaluated between 8 and 22 weeks of gestation and observed no significant increase in the risk of PTB in the group with asymptomatic BV. Another meta-analysis published by Brocklehurst et al. (31) included 21 randomized clinical trials involving 7847 pregnant women with BV. Screening for BV using clinical criteria or Gram and treatment of the condition eradicated the bacteria from the genital tract within 20 weeks (RR 0.20; 95%CI: 0.05-0.76) but did not reduce significantly the occurrence of childbirth at a gestational age of less than 37 weeks (RR 0.88; 95%CI: 0.71-1.09). Analysis of evidence from the literature and from the present study may suggest that asymptomatic BV detected between 20 and 25 weeks is not associated with PTB.

The present data demonstrated an association of other risk factors with spontaneous PTB: a history of PTB is a strong predictor of a new PTB in subsequent pregnancies, as previously reported by Esplin et al. (23). The crude and adjusted RR values were very high both among patients with 1 PPTB and those with 2 or more PPTB. This information was obtained with the prenatal questionnaire, which was applied by lay persons, and was only used by us during data processing. For this reason, even though this is a well-defined risk factor, these patients were not identified at the proper time for referral to reference centers specialized in prematurity. There was no association between maternal age and prematurity although literature data indicate a higher risk of PTB among women younger than 19 years and older than 35 years (24). The reference used here regarding parity was having 2 or 3 children. There was no increase in the risk of prematurity among primiparous patients and only an increase in crude RR was detected among them. This result supports data obtained by Bezerra et al. (22) in a study in which no association was detected between number of gestations and prematurity. Regarding the smoking variable, the crude risk of women that were smokers was similar to that reported in the literature by Grantz et al. (25).

In the present study, objective techniques were used for the measurement of the major exposures, with previous calibration of the instruments. The design of the study permitted the assessment of biological material of the pregnant women for the detection of infections. Although a specific sample calculation was not performed, with the total number of subjects in the initial thematic project (BRISA) being used to assess the association of factors related to PTB, it was observed that the sample size used had enough power for the estimate of associations of interest.

A limitation of the present study was that the patients were not monitored during the prenatal period, being evaluated only once prenatally and once after childbirth. Thus, there was no control of the number of pregnant women who had symptomatic UTI and BV throughout gestation and it was not known whether or not they had been treated. An additional limitation was that we did not know how the patients with a short cervical length had been followed up.

### Conclusions

In the present study, there was no association of asymptomatic UTI or BV with cervical shortening determined between 20 and 25 weeks of pregnancy. UTI, but not BV, was associated with spontaneous PTB. There was a relationship between early cervical shortening and prematurity, with a lower incidence than that reported in the literature.
